# Effectiveness of Preoperative Home Physiotherapy in Anterior Cruciate Ligament Deficiency

**DOI:** 10.7759/cureus.94439

**Published:** 2025-10-13

**Authors:** Nor Hamdam Fakru, Sahran Yahaya, Samhani Ismail, Nur Azree Ferdaus Kamudin, Wan Mahafeez Kamarul Zaman, Muzaffar T Shihabudin

**Affiliations:** 1 Department of Orthopaedics, Universiti Sains Malaysia School of Medical Sciences, Kota Bharu, MYS; 2 Department of Anatomy and Physiology, Faculty of Medicine, Universiti Sultan Zainal Abidin, Kuala Terengganu, MYS; 3 Department of Orthopaedics and Traumatology, Faculty of Medicine, Universiti Sultan Zainal Abidin, Kuala Terengganu, MYS; 4 Department of Orthopaedics and Traumatology, Hospital Pengajar Universiti Sultan Zainal Abidin, Kuala Terengganu, MYS; 5 Department of Orthopaedics, Universiti Sains Malaysia Specialist Hospital, Kota Bharu, MYS

**Keywords:** anterior cruciate ligament reconstruction, chronic acl deficiency, equipment-free physiotherapy, knee, knee stability, physical therapy modalities, rehabilitation

## Abstract

Anterior cruciate ligament (ACL) injury frequently compromises knee stability and function. Preoperative physiotherapy has been shown to improve surgical outcomes by enhancing periarticular muscle strength, mobility, and recovery. However, many existing protocols require exercise equipment and frequent supervision, limiting feasibility in resource-constrained settings. To date, no study has evaluated a simplified, equipment-free home-based physiotherapy program for chronic ACL deficiency. This prospective study assessed the effectiveness of a structured home-based physiotherapy program on 27 (male) patients with ACL deficiency awaiting reconstruction. The program focused on quadriceps and hamstring strengthening, range of motion, and proprioceptive training. Clinical outcomes were measured using the International Knee Documentation Committee (IKDC) score, Lysholm score, and knee range of motion at baseline and upon program completion. Participants demonstrated statistically significant improvements in quadriceps strength, IKDC and Lysholm scores, and range of motion compared with baseline (p < 0.05), along with reduced pain and improved knee stability. Adherence was high, and the program was well tolerated. These findings suggest that a structured, equipment-free home-based physiotherapy program is effective in improving function, strength, and mobility in patients with ACL-deficient knees. Incorporating such programs preoperatively may facilitate enhanced postoperative recovery and long-term knee function.

## Introduction

Anterior cruciate ligament (ACL) injury is among the most common ligament injuries, affecting approximately one in 3500 individuals, particularly young, active adults, with higher prevalence in females [1]. ACL reconstruction is one of the most frequently performed procedures in sports orthopaedics, with about 175,000 surgeries reported in the United States in 2000 at an estimated cost exceeding US$2 billion [2].

The ACL plays a critical role in preventing anterior tibial displacement, restraining internal rotation and valgus angulation, and providing proprioceptive sensation of knee joint positions [3]. ACL injury is functionally disabling, predisposing the knee to secondary injuries and early degenerative changes. Typical ACL tears often result from non-contact mechanisms such as a rapid deceleration, hyperextension, or rotational trauma. Characteristic features commonly are an audible ‘pop’, deep knee pain, and acute swelling due to hemarthrosis from rupture of the vascular ACL, often accompanied by inability to continue activity. In contrast, collateral ligament tears rarely produce swelling, while meniscal tears are more commonly associated with delayed swelling, occurring the following day [3-6].

Surgical reconstruction is typically indicated for patients with functional instability or those seeking to return to pivoting sports such as football, basketball, or badminton, while conservative management may be appropriate for less active individuals willing to modify their lifestyle. ACL reconstruction is typically performed using autografts or allografts, including patellar tendon autograft, hamstring tendon autograft, quadriceps tendon autograft, allograft patellar tendon, and cadaver graft [4]. Long-term success rates following ACL reconstruction range from 82% to 95%, although recurrent instability occurs in approximately 8% of patients [7]. 

Preoperative muscle strength greatly influences postoperative outcomes. Eitzen et al. reported that quadriceps strength prior to ACL reconstruction predicts knee function two years post-surgery [8]. Similarly, structured physiotherapy benefits non-operative patients by improving knee confidence, stability, and function [9]. Regardless of management, rehabilitation and physiotherapy remain essential to restore muscle strength, improve proprioception, and optimize joint stability [9]. However, frequent hospital-based physiotherapy visits may be inconvenient, due to distance, raising a need for a home-based physiotherapy approach, which offers a more practical and cost-effective alternative.

Grant et al. provided Level I evidence that a structured home-based rehabilitation program achieved better knee range of motion within the first three months after ACL reconstruction compared with standard care [10]. Rhim et al. reported greater short-term muscle strength gains with supervised rehabilitation compared to home-based training [11]. However, interpretation was limited by crossover between groups and differing program duration (six months versus six weeks). Keays et al. demonstrated that patients with chronic ACL deficiency achieved significant improvements in muscle strength, balance, passive stability, functional stability, agility, and function after six weeks of home-based physiotherapy [12]. However, their protocol required exercise equipment and frequent physiotherapist supervision, limiting feasibility in resource-constrained settings.

To date, studies evaluating a simplified, equipment-free home-based physiotherapy program for chronic ACL deficiency are scarce. This study aimed to evaluate the effectiveness of a newly developed, simplified home-based physiotherapy program for patients with chronic ACL deficiency. We hypothesized that the program would improve muscle strength and functional outcomes while providing a practical alternative to hospital-based rehabilitation. Positive findings may establish home-based physiotherapy as an effective alternative for both surgical and conservatively managed patients with ACL deficiency, reducing hospital visits, minimizing disruption to daily routines, and alleviating the burden on physiotherapists. Economically, fewer hospital visits may lower travel expenses, minimize work-related productivity loss, and decrease hospital costs associated with equipment procurement and maintenance.

## Materials and methods

This was a prospective interventional study conducted at the Orthopaedic Sports Clinic, Universiti Sains Malaysia Specialist Hospital (HPUSM), Kubang Kerian, Kelantan, Malaysia, between June and November 2013. The study was approved by the Human Research Ethics Committee, Universiti Sains Malaysia (registration number: USMKK/PPP/JEPeM|263.4/[1.8])

Participants

Patients aged 20-35 years with unilateral chronic ACL injury (>6 weeks), a positive anterior drawer or pivot-shift test, and subjective instability were eligible. Exclusion criteria included acute or bilateral ACL injury, multi-ligamentous knee instability, or prior ACL reconstruction. Concomitant meniscal injury was not an exclusion criterion.

Sample size

We used the following two-means formula (α = 0.05, β = 0.8, σ = 0.19, δ = 0.13) [1], to calculate the sample size: 

$n = \frac{2 \sigma^{2} \left( Z_{1-\alpha/2} + Z_{1-\beta} \right)^{2}}{\delta^{2}}$ 

where α is the significance level, β is the probability of a Type II error, δ is the effect size, and 𝜎 is the standard deviation of the population.

Based on this, the minimum sample size required was 19. Allowing for a 10% dropout rate, 27 patients were recruited consecutively.

Procedures

Baseline assessment included isokinetic muscle strength testing, knee stability evaluation, and functional scoring. Patients then received instruction from a physiotherapist on a structured six-week home-based physiotherapy program. A mid-program review at week 3 ensured correct technique and adherence. Final reassessment was performed at week 6.

Outcome measures

Outcome measures included: (i) Muscle strength: Quadriceps and hamstring strength were assessed using the Biodex Isokinetic Dynamometer (Biodex Medical Systems, Inc., New York, United States) at 180°/s (10 repetitions) and 300°/s (15 repetitions). Strength was expressed as the injured/uninjured limb peak torque ratio. (ii) Knee stability: Passive anterior tibial translation was measured using the KT-1000 arthrometer in 30° knee flexion. Three manual maximum displacement (MMD) measurements were performed per knee, with the highest recorded. Side-to-side differences were calculated. (iii) Functional outcome: Patient-reported knee function was assessed using the Lysholm Knee Score, administered by the investigator.

Home-based physiotherapy program

**Figure 1:**
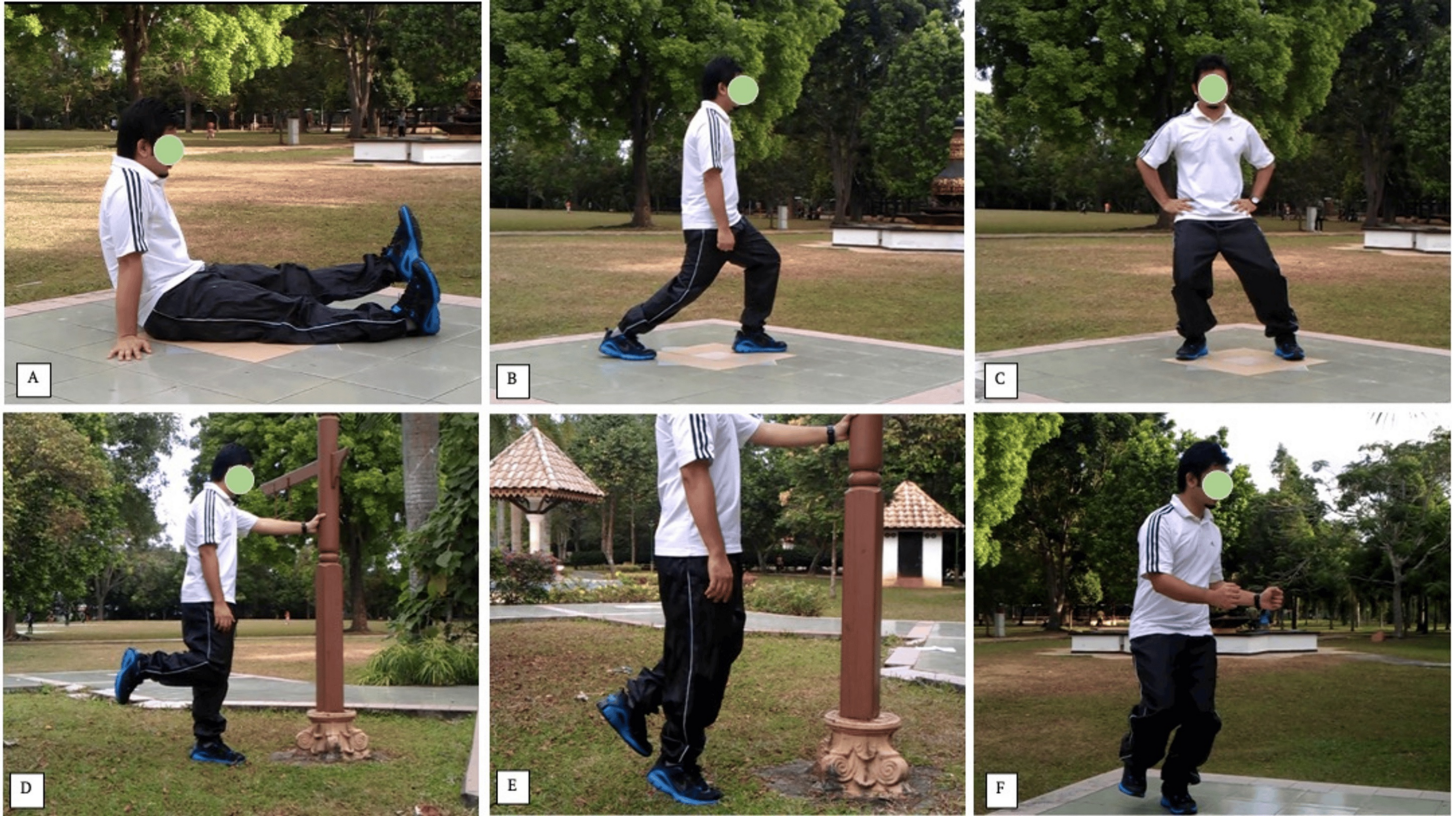
The home-based excercise protocol comprised: (A) Straight leg raise with knee at 0 degree with hamstring co-contracted, (B) Small lunges with hamstring co-contracted, (C) Split step stand on injured side, (D) Knee flexion in standing with well-controlled tibial rotation, (E) Single-leg calf raise with knee in extension, (F) Turnaround control – small arc knee bends, alternating between internal and external rotation. Our exercise protocol was adapted from Keays et al. [6] and simplified to exclude equipment

The exercise program, adapted from Keays et al. [12] and simplified to exclude equipment, consisted of quadriceps strengthening through straight leg raises (30-50 repetitions), small lunges (10-20 repetitions), and split-step stand (20-50 repetitions). Hamstring strengthening was performed using standing knee flexion with controlled tibial rotation (10-30 repetitions), while calf strengthening involved single-leg calf raises in extension (10-20 repetitions). Proprioceptive training was incorporated through small-arc knee bends with alternating internal and external rotation (Figure 1).

Statistical analysis

Data were analyzed using IBM SPSS Statistics for Windows, version 20 (IBM Corp., Armonk, New York, United States). Paired-samples t-tests were applied to compare pre- and post-intervention muscle strength ratios, knee stability, and Lysholm scores. Non-normally distributed data were analyzed using the Wilcoxon signed-rank test, while Pearson correlation was used to assess relationships between muscle strength, functional scores, and knee stability. Statistical significance was set at p <0.05.

## Results

Study population

Of the 27 patients enrolled, 20 completed the six-week home-based physiotherapy program and were included in the analysis. All were male, with a mean age of 26.1 years. Most injuries occurred during football (n = 11) or futsal (n = 8), while one was sustained during hockey. The mean interval between injury and initiation of physiotherapy was 16.8 weeks. ACL injury grades were: Grade I (n = 6), Grade II (n = 10), and Grade III (n = 4). Demographic and clinical characteristics were tabulated in Table 1.

**Table 1:** Demographic and clinical characteristics of participants (N = 20) Data presented as n (%) and mean

Variable	Frequency (Percentage)
Age (years), mean	26.1
Male Sex	20 (100%)
Injury mechanism	
Field football	11 (55%)
Futsal	8 (40%)
Hockey	1 (5%)
Injury-to-physiotherapy interval (weeks), mean	16.8
ACL injury grade	
Grade I	6 (30%)
Grade II	10 (50%)
Grade III	4 (20%)

Muscle strength

The mean quadriceps strength ratio (injured/uninjured side) at 180°/s improved from 0.72 pre-physiotherapy to 0.95 post-physiotherapy, with a mean difference of 0.23 (p < 0.001). Similarly, the mean hamstring strength ratio increased from 0.80 to 1.02 at 180°/s (p < 0.001). At 300°/s, the quadriceps strength ratio improved from a median of 0.75 to 0.97 (p < 0.001), while the mean hamstring strength ratio increased from 0.86 to 1.01 (p < 0.001). Overall, both quadriceps and hamstring strength ratios significantly increased at both testing velocities following six weeks of home-based physiotherapy. Table 2 shows the changes in muscle strength prior to and after physiotherapy sessions.

**Table 2:** Changes in muscle strength ratios (injured/uninjured limb) prior and upon physiotherapy.

Variable (°/s)	Pre-physiotherapy	Post-physiotherapy	Mean difference (p value)
Quadriceps 180	0.72 (0.17)	0.95 (0.11)	0.23 <0.001*
Hamstring 180	0.80 (0.13)	1.02 (0.16)	0.22 <0.001*
Quadriceps 300	0.75 (0.12)	0.97 (0.15)	0.22 <0.001*
Hamstring 300	0.86 (0.11)	1.01 (0.11)	0.15 <0.001*

Knee stability

No significant difference was found in passive knee stability (KT-1000 arthrometer). Results from Pearson Correlation also showed no significant difference in the relationship between the stability of the quadriceps or hamstrings at 180 or 300 degrees.

## Discussion

ACL injury is one of the most common knee injuries encountered in orthopaedic practice, and rehabilitation plays an integral role in both conservative and operative management. Patients with chronic ACL deficiency often present with a good range of motion but persistent muscle weakness and functional instability [13]. This study assessed the effectiveness of a simplified, equipment-free home-based physiotherapy program in improving muscle strength, knee function, and stability in this group of patients.

In terms of muscle strength, our findings demonstrated significant improvements in both quadriceps and hamstring strength following the program. Consistent with previous reports, patients initially exhibited marked strength deficits in the injured limb compared to the contralateral side [8,14]. After six weeks, the injured-to-uninjured strength ratio exceeded 0.9, surpassing the commonly recommended threshold of 0.8 prior to ACL reconstruction [7]. Importantly, bilateral gains were observed, reflecting the benefit of exercises performed on both limbs. These results highlight the ability of a simple, progressive home program to restore strength effectively without requiring specialized equipment.

For passive knee stability evaluation, no significant improvement was observed in passive anterior tibial translation, as measured by the KT-1000 arthrometer. This aligns with previous studies indicating that physiotherapy enhances dynamic stability rather than passive stability. Keays et al. reported improvements in KT-1000 measurements following a more intensive program; however, their findings have not been consistently reproduced [12]. In our study, the mean anterior translation of the tibia in relation to the femur was 7.75 mm before physiotherapy and 7.70 mm after, with no statistically significant difference. This result was expected, as the KT-1000 assesses passive restraints only. Physiotherapy primarily strengthens the dynamic stabilizer against anterior tibial translation, which was injured in all participants. Since exercise cannot directly restore ACL integrity [5], improvements in passive knee stability were not observed. Thus, unlike Keays et al. [12], we could not demonstrate gains in KT-1000 measurements following physiotherapy. Instead, our findings are consistent with the broader literature, which shows that physical exercise does not improve passive stability in ACL-deficient knees [15].

Patient-reported function, as assessed with the Lysholm Knee Score, improved significantly from a mean value of 69.5 to 80 following the home-based physiotherapy. It assessed patients’ function and condition across domains such as limping, walking support, stair climbing, squatting, dynamic instability, swelling, pain, and thigh atrophy. In our cohort, improvements were most notable in dynamic instability and pain, which likely explain the overall functional gains despite unchanged passive stability. This suggests that enhanced muscular support and proprioception contribute meaningfully to patient-perceived stability during daily activities.

Regarding compliance, 20 participants completed the program, corresponding to a 30% dropout rate. Reasons for dropout included distance from the hospital and unspecified factors. Nevertheless, adherence among those who completed the program was high, with favorable outcomes. Although home-based physiotherapy offers clear advantages in convenience, time, and cost, maintaining patient motivation and compliance remains more challenging than in supervised rehabilitation. To our knowledge, the strength of this study lies in its pragmatic design and robust evaluation, with the program adapted into practical yet effective protocols that encourage participation in resource-limited settings. Furthermore, this home-based physiotherapy model requires minimal supervision.

This study has several limitations. The patients’ activity levels or occupational levels were not taken into account, although they may influence the functional scores. Patients engaged in high-level sports may report lower subjective function compared to recreational athletes, who dominated our cohort. Another limitation is that ACL injury grades were not classified. Differences in improvement across subgroups could not be meaningfully analysed due to the small sample size. In addition, no female participants were included, reflecting the recruitment population but introducing potential gender bias and limiting generalizability. Finally, due to time constraints, long-term outcomes could not be evaluated. Future prospective studies with larger, more diverse cohorts and extended follow-up are required to clarify the durability of improvements, the impact of injury grade and sex, and the potential role of home-based physiotherapy in influencing surgical outcomes. This study demonstrates that a structured, equipment-free home-based physiotherapy program can significantly improve muscle strength and functional outcomes in patients with chronic ACL deficiency. Although passive knee stability remained unchanged, the observed improvements in dynamic stability and patient function underscore the role of pre-operative rehabilitation in optimizing recovery. Integration of such home programs into standard practice may enhance surgical outcomes while reducing the burden on hospital resources.

Home-based physiotherapy may serve as a valuable option for patients managed conservatively or as preoperative preparation prior to ACL reconstruction. Careful patient selection remains important, as those with acute injuries or associated multi-ligamentous involvement may require alternative approaches. Adherence is a critical factor, and further studies are needed to compare compliance and long-term outcomes of home-based versus hospital-based physiotherapy. Future research should also assess whether preoperative home programs can enhance surgical results or potentially reduce the need for reconstruction in selected patients.

## Conclusions

This prospective interventional study demonstrates that a simplified six-week home-based physiotherapy program is effective in improving quadriceps and hamstring strength, as well as functional outcomes, in patients with chronic ACL deficiency. However, this program could not alter the passive knee stability; therefore, later surgical intervention may still be required.
